# Combined Effects of Environmental and Lifestyle Exposures on Liver Health: The Mediating Role of Allostatic Load

**DOI:** 10.3390/toxics13110935

**Published:** 2025-10-30

**Authors:** Esther Ogundipe, Emmanuel Obeng-Gyasi

**Affiliations:** 1 Department of Built Environment, North Carolina A&T State University, Greensboro, NC 27411, USA; eoogundipe@aggies.ncat.edu; 2 Environmental Health and Disease Laboratory, North Carolina A&T State University, Greensboro, NC 27411, USA

**Keywords:** allostatic load, liver diseases, heavy metals, lead, cadmium, mercury, smoking, Alcohol Drinking, physiological stress, fatty liver index, Bayesian Kernel Machine Regression, Causal Mediation Analysis, NHANES

## Abstract

***Background:*** Liver disease is a growing global health burden. While individual environmental exposures like heavy metals (lead, cadmium, mercury) and behavioral factors such as smoking and alcohol use are known risk factors, their combined impact and the underlying physiological pathways are poorly understood. Allostatic load (AL), a measure of cumulative physiological stress, is a potential mediator or modifier in the relationship between these chronic exposures and liver disease. This study aimed to investigate the joint effects of heavy metals and behavioral exposures on liver health and to examine the mediating role of AL. ***Methods:*** This cross-sectional study utilized data from the National Health and Nutrition Examination Survey (NHANES) 2017–2018 cycle. We assessed blood concentrations of the environmental and lifestyle variables in relation to liver biomarkers and the Fatty Liver Index (FLI). Descriptive statistics were used to summarize participant characteristics. Multivariable linear regression and Bayesian Kernel Machine Regression–Causal Mediation Analysis (BKMR-CMA) were used to model combined, nonlinear effects of the exposure–outcome mixture and to evaluate the mediating role of AL. ***Results:*** Lead exposure was positively associated with higher AST (β = 0.65, *p* = 0.04) and GGT (β = 1.99, *p* = 0.05), while smoking increased GGT (β = 0.79, *p* = 0.03) and ALP (β = 0.78, *p* < 0.01). AL independently predicted higher FLI (β = 3.66, *p* < 0.001). ***Conclusions:*** This study highlights that liver health is influenced by the combined effects of environmental pollutants, behaviors, and cumulative biological stress. While lead exposure and smoking were independently linked to liver enzyme elevations, and AL to FLI, mediation by AL was limited, though trends suggest AL may still amplify chronic metabolic pathways leading to liver disease.

## 1. Introduction

Liver disease represents a significant and growing burden on global health, with far-reaching consequences for individuals, families, and healthcare systems [1]. Liver health can be assessed through key enzymes and factors such as Aspartate Aminotransferase (AST), Alanine Aminotransferase (ALT), Gamma-Glutamyl Transferase (GGT), Alkaline Phosphatase (ALP), and Total Bilirubin [2]. AST and ALT are critical markers used to detect hepatocellular injury, with elevated levels indicating liver cell damage and help to differentiate the severity of liver injury [2,3]. GGT and ALP serve as markers for bile duct dysfunction or cholestasis. When ALP is elevated, a rise in GGT confirms it is coming from the liver, especially in cases like obstructive or infiltrative liver diseases. If GGT is normal, the cause is likely non-hepatic, such as a bone issue [4,5]. Total Bilirubin serves as a measure of hepatic excretory capacity and bile metabolism [2]. Distinctions between bilirubin fractions yield additional diagnostic information: conjugated (direct) hyperbilirubinemia generally indicates intrahepatic or extrahepatic obstruction or hepatocellular dysfunction, whereas unconjugated (indirect) hyperbilirubinemia suggests hemolysis or impaired hepatic uptake and conjugation [2,5]. Taken together, these enzymes and metabolites provide an integrated assessment of hepatic function and pathology, offering valuable diagnostic and prognostic information across a spectrum of liver diseases. As key biomarkers, they capture diverse aspects of liver health, ranging from hepatocellular injury and cholestasis to disturbances in bile metabolism, thereby serving as essential tools for both clinical evaluation and population-based studies of hepatic outcomes.

Liver disease causes over two million deaths annually worldwide, accounting for approximately 4% of all global fatalities. From cirrhosis and hepatocellular carcinoma to metabolic dysfunction-associated fatty liver disease (MAFLD), originally referred to as non-alcoholic fatty liver disease (NAFLD), liver dysfunction poses immense health challenges, exacerbated by socioeconomic costs that strain public health resources [6,7,8]. This escalating burden highlights the urgent need to identify and mitigate modifiable risk factors to reduce liver disease incidence and improve population health outcomes.

Among the numerous contributors to liver disease, environmental and behavioral exposures stand out as major yet understudied risk factors. Heavy metals such as lead, cadmium, and mercury, found in industrialized environments, are well-documented toxicants that can impair liver function by causing oxidative stress, inflammation, and hepatocellular damage [9,10]. Concurrently, behavioral factors such as smoking and excessive alcohol consumption are well-established causes of liver dysfunction, particularly in vulnerable populations [11,12]. These exposures are not mutually exclusive; new research suggests that their combined effect may exacerbate liver damage, increasing the risk of disease progression [13].

Despite rising awareness, the interplay between environmental and behavioral factors is still poorly understood, particularly how these exposures lead to liver disease through complicated physiological systems [14]. This research seeks to investigate the combined influence of metals and behavioral exposures on liver health, with a focus on allostatic load (AL), which is a comprehensive biomarker of chronic stress and physiological dysregulation as a potential mediating factor. Understanding these connections could provide vital insights into the etiology of liver disease and enable focused prevention methods, thereby lowering the worldwide burden of this widespread health problem.

The concept of AL is fundamental to comprehending the relationship between chronic exposures and liver disease. AL represents the cumulative biological “wear and tear” that arises from chronic activation of the body’s stress response systems over time [15,16]. AL helps explain how long-term exposure to physiological stressors including environmental pollutants, unhealthy behaviors, and psychosocial stress can dysregulate multiple systems, including the neuroendocrine, immune, and metabolic pathways and lead to physical changes in the body that cause disease [13,17]. Previous studies have shown how AL exposure can cause detrimental effects on the neuroendocrine system, leading to metabolic syndrome (MetS), which is closely linked to liver health [18]. This dysregulation is measurable through biomarkers that collectively reflect the body’s adaptive load over time [19]. AL is not merely a byproduct of stress; it is also an active measure of how accumulated stressors translate into long-term health outcomes. Research has linked elevated AL to numerous chronic conditions, including cardiovascular diseases, diabetes, and new research suggests it may also play a role in liver disease [20,21].

Chronic stress, operationalized as AL, may exacerbate the hepatotoxic effects of exposures to heavy metals and harmful behaviors, intensifying oxidative stress, inflammation, and cellular damage within the liver [13,20]. It may essentially act as a mediating factor, showing how these external factors translate into internal health problems. When it comes to liver disease, AL highlights how chronic stress can worsen the effects of harmful exposures, like heavy metals or smoking. This makes AL a potential valuable way to understand the complex connections between stress, behavior, and liver health, giving us a new perspective on what drives liver disease [18,22].

The combined effects of heavy metals and behavioral factors on liver health are likely to extend beyond their individual contributions. A synergistic interaction may occur, wherein the concurrent presence of these risk factors amplifies their negative impact on liver function [9,10]. For instance, exposure to heavy metals may compromise the liver’s ability to detoxify alcohol or metabolize nicotine, compounding the harm caused by behavioral factors [23,24]. Similarly, smoking or alcohol consumption may exacerbate the oxidative stress and inflammatory responses triggered by metal toxicity [13].

AL emerges as a potential pivotal element of the exposure–response system, offering a methodical explanation for these synergistic effects. By integrating concepts from environmental toxicity and behavioral factors, this research hypothesizes that AL amplifies the health impacts of these combined exposures through multi-pathway interactions. The stress-response system positions AL not only as an outcome of cumulative stressors but also as a cause of downstream health effects, such as liver disease [25]. Investigating this interconnected system could yield comprehensive insights, providing preventative strategies that address these multiple risk factors altogether.

**Research Gap and Rationale for the Study**

Despite the extensive research conducted on liver disease, attention has been focused on individual risk factors such as heavy metals like lead, mercury, and cadmium or behavioral exposures like smoking and alcohol use, leaving the combined impact of these factors unexplored. Previous research often works in isolation, ignoring the possibility of a compounding or synergistic interaction between environmental and behavioral risks. Furthermore, the physiological pathways linking these exposures to liver health problems are poorly understood. Also, AL, which is a measure of biological stress, has not been considered as a mediator. This gap is noteworthy because AL provides a unique perspective to understand how chronic stress may amplify the negative impact of these exposures on liver health [26]. This research aims to check if AL is a critical link between these risk factors and liver disease.

**Study Objective and Framework**

The objective of this study is to examine the joint influence of heavy metal exposures and behavioral factors on liver disease, with particular attention to the mediating role of AL. By integrating environmental exposures, behavioral determinants, and stress physiology, this work seeks to elucidate how these interconnected factors combine to shape liver health.

## 2. Materials and Methods

### 2.1. Study Design

This study utilized data from the National Health and Nutrition Examination Survey (NHANES) for the years 2017–2018. NHANES is a cross-sectional survey designed to assess the health and nutritional status of a nationally representative sample of the non-institutionalized U.S. population residing in the United States [27]. Conducted by the U.S. Centers for Disease Control and Prevention (CDC), the survey employs a stratified, multi-stage and clustered sampling design to ensure representativeness of the U.S. population. Data collection includes demographic information, physical examinations, and laboratory analyses, all approved by the Institutional Review Board of the National Center for Health Statistics (NCHS; Hyattsville, MD, USA). The demographic data was collected through household interviews using a Computer-Assisted Personal Interview (CAPI) system (Westat, Inc., Rockville, MD, USA). Participants provided information on age, sex, ethnicity, income, smoking status, alcohol consumption, and body mass index (BMI). These data were collected in multiple languages to ensure inclusiveness and accuracy. Informed consent was obtained from all participants before participation. The survey protocols were approved by the Institutional Review Board at the National Center for Health Statistics, CDC.

### 2.2. Variables and Covariates, Mediators, and Effect Modifiers

#### 2.2.1. Variables and Covariates

The primary outcome variable was liver health, assessed using established biochemical markers including AST, ALT, GGT, ALP, total bilirubin, and the FLI. Predictor variables consisted of blood concentrations of Pb, Cd, and Hg, measured in micrograms per deciliter (µg/dL).

Behavioral covariates were derived from NHANES questionnaires. Smoking status was determined from variable SMD650 (“Average number of cigarettes per day during the past 30 days”), which asked participants: “During the past 30 days, on the days that you smoked, about how many cigarettes did you smoke per day?” Responses ranged from 1 to 95 cigarettes per day, with one pack defined as 20 cigarettes. Alcohol consumption was assessed using variable ALQ142 (“Number of days having four or five drinks in the past 12 months”), which inquired: “During the past 12 months, about how often did you have four (for females) or five (for males) or more drinks of any alcoholic beverage in a single day?” Responses ranged from “never in the last year” to “every day.”

Sociodemographic and anthropometric covariates included age (years), sex, race/ethnicity, education level, household income, and body mass index (BMI; kg/m^2^). These covariates were selected a priori based on their established associations with liver function and potential to confound the relationships between metal exposure, behavioral factors, and hepatic outcomes.

#### 2.2.2. Mediators and Effect Modifiers

AL was examined as a potential mediating variable. Consistent with established indices used in NHANES-based studies, AL was derived from ten physiological biomarkers representing cardiovascular (systolic and diastolic blood pressure, triglycerides, HDL cholesterol, total cholesterol), metabolic (body mass index, hemoglobin A1c, serum albumin, creatinine clearance), and inflammatory (C-reactive protein) systems. Each biomarker was dichotomized into high-risk (1) or low-risk (0) categories based on clinical reference values or quartile distributions. For most biomarkers, values in the upper 25% of the distribution were categorized as high risk; however, for albumin, creatinine clearance, and HDL cholesterol, the lower 25% represented elevated risk. Summing up, these binary indicators produced a composite AL score ranging from 0 to 10. For descriptive analyses, scores ≤ 3 were classified as low AL and scores > 3 as high AL. Age was modeled as a continuous variable and considered an effect modifier, with evaluations conducted at selected percentiles (10th, 30th, 70th, and 90th) to capture potential differences across the life course.

### 2.3. Inclusion and Exclusion Criteria

Participants aged 18–80 years with complete data on heavy metal exposures, behavioral factors, FLI, and AL biomarkers were included in the analysis. Individuals with missing information for any of these variables were excluded. This approach inherently excluded participants, such as pregnant women, for whom these laboratory measurements were not collected in the NHANES 2017–2018 cycle.

### 2.4. Measurement of Heavy Metals (Lead, Cadmium, and Mercury)

The levels of heavy metals in whole blood samples were measured using inductively coupled plasma dynamic reaction cell-mass spectrometry (ICP-DRC-MS; PerkinElmer, Inc., Waltham, MA, USA). These assays were conducted by the CDC’s Division of Laboratory Sciences at the National Center for Environmental Health. Detailed laboratory procedures regarding quality control and quality assurance can be found on the NHANES website (Survey, 2017–2018).

### 2.5. Alcohol and Smoking Assessment

Alcohol consumption was assessed based on the NHANES Alcohol Use Questionnaire, which includes data on both lifetime and current alcohol use. Smoking behavior, on the other hand, was evaluated using data on cigarette use, with the focus on current smoking status (NHANES, 2017–2018).

### 2.6. Statistical Analysis

#### 2.6.1. Descriptive Statistics and Regression Analysis

Descriptive statistics were performed to summarize participant characteristics, liver enzyme levels, metal exposures, and behavioral factors. Spearman correlation analysis was conducted to explore relationships among study variables, including liver biomarkers, metals, behavioral exposures, and demographic factors. Univariate and multivariable linear regression models were employed to assess associations between predictor variables (lead, cadmium, mercury, smoking, and alcohol consumption) and the outcome (FLI). Covariates used for model adjustment included age, sex, BMI, ethnicity, and income. Complete data was used for the analysis.

#### 2.6.2. Bayesian Kernel Machine Regression (BKMR)

BKMR was utilized to assess the combined impact of lead, cadmium, mercury, smoking, and alcohol consumption on FLI. BKMR is a flexible, nonlinear regression method that accommodates the detection of interaction effect among exposures and predictors. Unlike traditional linear regression models, BKMR can model complex, nonlinear relationships between contaminants and health outcomes. The BKMR model structure can be expressed as:

$$Y=\;\beta_{0}+h\left(X\right)+\beta^{T}C+\;\epsilon$$

where Y represents the outcome (Fatty Liver Index), β is the intercept, $h\left(X\right)$ represents the exposure–response functions for the contaminants, $\beta^{T}$ are the regression coefficients for covariates, C denotes the confounders, and ϵ is the random error term. Exposure–response functions were modeled nonparametrically using Gaussian kernels. Markov Chain Monte Carlo (MCMC) sampling was used to estimate model parameters and exposure–response relationships, with a total of 50,000 iterations to ensure convergence.

Statistical analysis was conducted using R software (version 4.2.3; R Foundation for Statistical Computing, Vienna, Austria), and a significance level of 0.05 was used for non-Bayesian analyses.

#### 2.6.3. Bayesian Kernel Machine Regression Causal Mediation Analysis (BKMR-CMA)

Bayesian Kernel Machine Regression–Causal Mediation Analysis (BKMR-CMA) was applied to investigate the mediating role of AL in the relationship between metal exposures, behavioral factors, and liver health. This method allows for the estimation of natural direct effects (NDE) and natural indirect effects (NIE) while accounting for nonlinear and interactive exposure effects. We used BKMR to model both the mediator and outcome relationships. The mediator model is represented by:

$$M_{i}=\;h_{M}\left(Z_{Mi}\right)+\;C_{i}^{T}\beta +\;\epsilon_{Mi}$$

where $M_{i}$ is the mediator (AL), $Z_{Mi}$ represents exposure variables (metals and behavioral factors), $C_{i}$ denotes covariates, $h_{M}(.)$ is a flexible function capturing nonlinear relationships, and $\epsilon_{Mi}\sim N(0,\sigma^{2}M)$ is an error term.

The outcome model is denoted by:

$$Y_{i}=\;h_{Y}\left(Z_{Yi},M_{i}\right)+\;C_{i}^{T}\theta +\;\epsilon_{Yi}$$

where $Y_{i}$ represents liver health outcomes, $Z_{Yi}$ includes exposure and behavioral factors, $M_{i}$ is the mediator (AL), and $h_{Y}(.)$ is a flexible function modeling nonlinear and interaction effects.

The total effect model is represented by $Y_{i}=\;h_{TE}\left(Z_{Yi}\right)+\;C_{i}^{T}\gamma +\;\epsilon_{TEi}$ where $h_{TE}(.)$ models the total effect of exposures on liver health. The Causal Mediation Analysis (CMA) effects were estimated using the following counterfactual-based definitions:

Total effect, *TE*:

$$TE=E[Y_{a}-Y_{a*}]$$

Natural direct effect, *NDE*:

$$NDE=E[Y_{a}M_{a*}-Y_{a*}M_{a*}]$$

Natural indirect effect, *NIE*:

$$NIE=E[Y_{a}M_{a*}-Y_{a}M_{a*}]$$

Controlled direct effect, *CDE*:

$$CDE\left(m\right)=E[Y_{am}-Y_{a*m}]$$

where $Y_{a}M_{a*}$ represents the counterfactual outcome when the exposure is set to a, but the mediator is fixed at the level it would have taken under $a^{*}$.

Age was modeled as a continuous variable and evaluated at selected percentiles to capture life-course variations in exposure effects: the 10th percentile (younger adults), 30th percentile (mid-age adults), 70th percentile (late mid-age adults), and 90th percentile (older adults). These percentiles cut points were chosen to represent distinct life stages while maintaining adequate sample size within each group. Age functioned as an effect modifier in the BKMR-CMA framework, allowing assessment of whether the estimated direct and indirect effects differed across age groups. All statistical analyses were conducted in R (version 4.4.1). For non-Bayesian procedures, a two-sided significance threshold of α = 0.05 was applied.

## 3. Results

### 3.1. Descriptive Statistics of Study Sample

A total of 308 participants were included in this study, with a breakdown of heavy metals (lead, cadmium, mercury) and AL across age deciles. Given the study’s focus on life-course variation and effect modification by age, the descriptive characteristics of the sample are presented in a stratified format rather than as a single aggregated table. This approach highlights differences in metal exposures, behavioral factors, and AL across age, sex, and race/ethnicity groups—patterns that are central to the interpretation of subsequent regression and mediation analyses. Stratified presentation allows clearer visualization of exposure trends and stress-related physiological differences across demographic subgroups, thereby aligning with the study’s analytical framework. In Table 1 below, Lead levels showed a progressive increase with age, with a peak in the 70–80 and 80–90 age groups with means of 3.05 and 2.71 µg/dL, respectively.

In contrast, cadmium levels (Table 2) remained relatively stable across age groups, though younger participants ages 10–20 and 30–40 displayed higher variability and slightly elevated mean levels, which may be due to early-life exposure or lifestyle factors.

**Table 3:** Mercury by Age Decile.

Age Decile (Years)	Mean (µg/dL)	Std Dev	Min	Max	Count
10–20	0.2	0.0	0.2	0.2	5
20–30	0.67	1.27	0.2	8.44	47
30–40	0.68	0.70	0.2	4.86	62
40–50	1.36	2.09	0.2	11.55	55
50–60	0.98	1.25	0.2	9.16	68
60–70	1.44	2.86	0.2	20.53	54
70–80	0.94	0.63	0.2	2.14	13
80–90	0.89	0.98	0.2	2.34	4

AL was elevated across all age groups, with most means ranging between 3.0 and 3.8. The highest AL was recorded in the 50–60 age bracket (mean = 3.82), possibly reflecting the accumulation of chronic stressors during midlife. The results are presented in Table 4 below.

**Table 4:** Allostatic Load by Age Decile.

Age Decile (Years)	Mean	Std Dev	Min	Max	Count
10–20	3.8	0.84	3.0	5.0	5
20–30	3.36	1.48	1.0	7.0	47
30–40	3.56	1.43	1.0	7.0	62
40–50	3.73	1.66	0.0	7.0	55
50–60	3.82	1.83	0.0	8.0	68
60–70	3.65	1.52	0.0	7.0	54
70–80	3.0	1.96	0.0	6.0	13
80–90	3.0	1.63	1.0	5.0	4

As shown in Table 5, the variable is divided into sex and race/ethnicity by levels of AL, categorized as either low (≤3) or high (>3). Among the females in this study, 53.23% had a high AL, compared to 46.77% with a low AL, suggesting a slightly higher burden of chronic stress. In contrast, 55.98% of males had a low AL, while 44.02% had a high AL This indicates that the males in this study were somewhat more likely to have lower AL than females. When considering race and ethnicity, Mexican Americans exhibited the highest proportion of high AL, with 73.33% falling in that category. In comparison, 65.69% of Non-Hispanic Black individuals had low AL, indicating a lower stress burden relative to other groups.

### 3.2. Linear Regression Results

As shown in Table 6 below, the linear regression was used to evaluate the independent associations of heavy metals (lead, cadmium, mercury), behavioral factors (alcohol consumption and smoking), and AL with liver enzyme biomarkers and FLI, while adjusting for key confounders including age, sex, BMI, income, education and ethnicity. For AST, lead exposure showed a statistically significant positive association (*p* = 0.04), whereas cadmium, mercury, alcohol, smoking, AL, and other demographic factors did not exhibit statistically significant associations.

For ALT, lead showed a marginally significant positive association, suggesting a potential link between lead exposure and liver stress. Body Mass Index (BMI) emerged as a strong and significant predictor (*p* = 0.01), and gender was also significantly associated with ALT, with *p* < 0.01. The results are shown in Table 7 below.

On the other hand, for ALP, both smoking (*p* < 0.01) and age (*p* < 0.01) were positively associated, while alcohol consumption and AL were not statistically significant but approached significance. The results are shown in Table 8 below.

In the case of GGT, both lead and smoking showed statistically significant positive associations, supporting their role in promoting oxidative stress and hepatotoxicity, while age was marginally significant (*p* = 0.05). The results are shown in Table 9 below.

Total Bilirubin (Table 10) showed that smoking and gender had significant negative associations. AL also approached significance.

Finally, for FLI (Table 11), AL was highly significant and positively associated (*p* < 0.001) while alcohol consumption was inversely associated with FLI. Age, gender, and BMI were all statistically significant predictors. Mercury approached significance (*p* = 0.08).

### 3.3. Assessing Relationships Among Key Variables: Spearman Correlation Matrix Analysis

**Figure 1:**
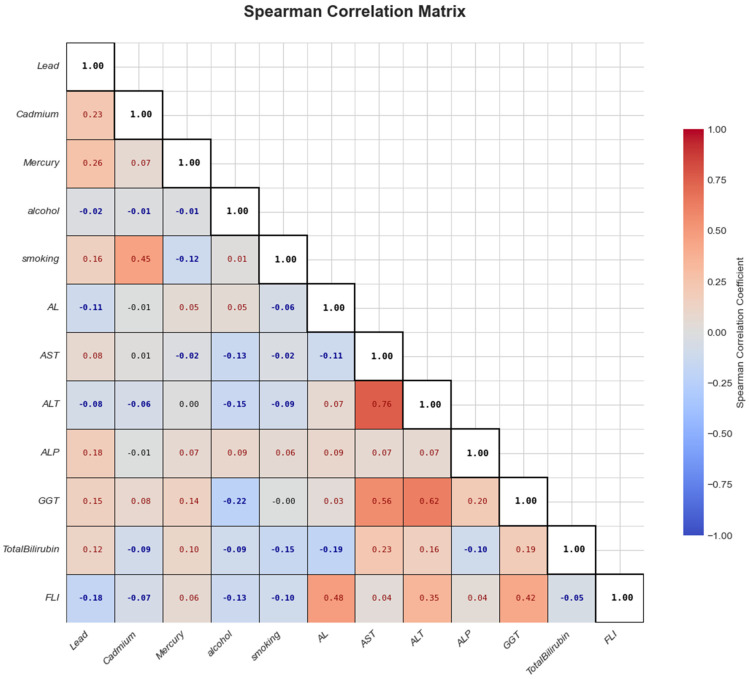
Spearman correlation plot of critical study variables.

Figure 1 illustrates the Spearman correlation analysis conducted among the key variables, that is, heavy metals, behavioral factors, AL, and liver biomarkers. Stronger correlations are shown with deeper colors—red for positive and blue for negative associations. From the analysis, AL had weak positive correlations with most liver biomarkers.

### 3.4. Risk Summary for Total Effects

**Figure 2:**
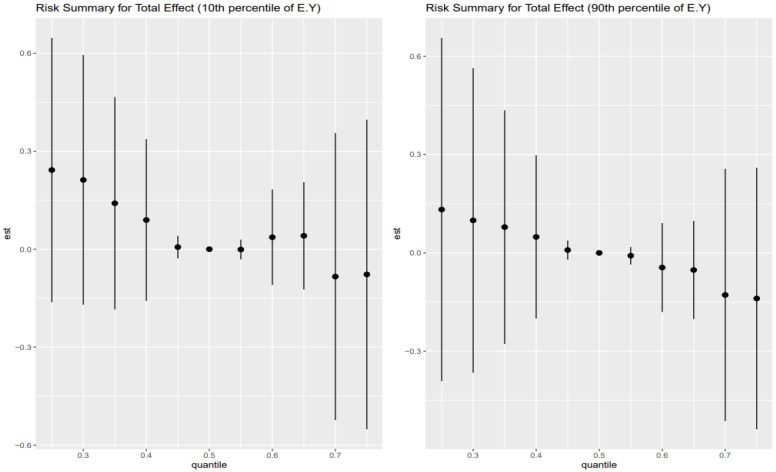
The overall exposure effect at the 10th and 90th percentiles of the combined mixture (metals and behavioral exposures) on the outcome (FLI) was examined at 0.25–0.75 quantiles of exposure as compared to the 0.5 quantile.

The Combined Impact of Heavy Metals and Behavioral Exposures on Liver Disease, as analyzed using BKMR, revealed the following (Figure 2):

Left Plot: 10th Percentile of Age (Younger Age Group)

Effect Estimates: The effect estimates (est) show the quantiles moving above zero, then fall back to zero between 0.45 and 0.55, then slightly go up but fall below zero as the quantiles increase.

Trend: There is a slight positive increasing trend across the lower exposure quantiles for younger individuals, at mid age, the effect estimates stay at zero, and slightly negative at higher quantiles.

Credible Intervals: The credible intervals are relatively wide, indicating uncertainty in the estimates. This suggests that Heavy metals and behavioral factors have an uncertain effect on FLI for younger age groups.

Right Plot: 90th Percentile of Age (Older Age Group)

Effect Estimates: For older age groups, the effect estimates move slightly above zero at lower quantile levels and zero at the mid quantile level. The effect estimates show a downward trend as the quantile goes higher.

Trends: As quantiles increase, there is a significant decrease in the effect estimates, suggesting that for older individuals, lower exposure levels might be associated with decrease in the FLI.

Credible Intervals: Just like the younger age group, the credible intervals are also wide for the older age group, showing a degree of uncertainty.

**Figure 3:**
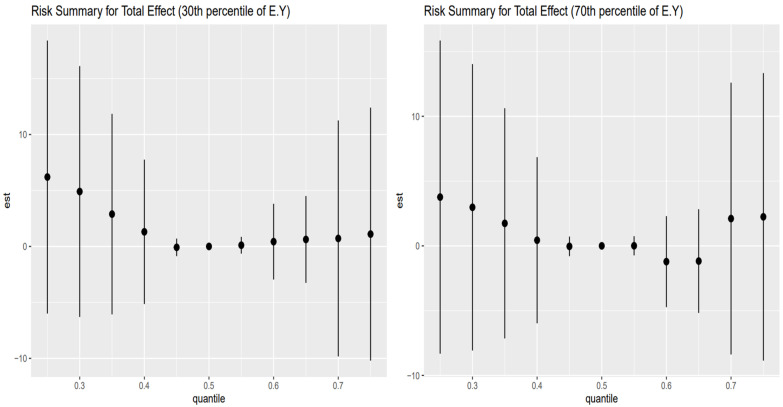
The overall exposure effect at the 30th and 70th percentiles for age, assessing the combined mixture (metals and behavioral exposures) on the outcome (FLI), was examined at 0.25–0.75 quantiles of exposure as compared to the 0.5 quantile.

The results for the 30th and 70th percentile are shown in Figure 3.

Left Plot: 30th percentile of Age (mid-age group)

Effect Estimates: The effect estimates show a decreasing trend as the exposure quantiles increase, moving from positive and then falling to zero as it approaches the mid quantile, and slightly increasing as the quantiles increase.

Trends: There is a mild increase in the estimated effect at higher quantiles, suggesting that for mid-younger individuals, high exposure levels may be associated with a slight increase in FLI, although the effect remains small.

Credible intervals: The credible intervals are wide, particularly at higher quantiles, showing a high degree of uncertainty.

Right Plot: 70th percentile of Age (late mid-age group)

Effect Estimates: The effect estimate is slightly above zero and positive as the quantiles increase and decrease below zero and then goes slightly upward again.

Trends: The trend for this age group fluctuates, shows a positive estimate and decreases as the quantiles increase, then approaches zero at the mid quantile, then the estimate becomes negative as the quantile increases, and slightly goes above zero and becomes positive at the highest quantile level. Credible intervals: The wide credible intervals consistently crossing zero across all quantiles indicate substantial uncertainty in the estimates.

### 3.5. Controlled Direct Effect Risk Summary

**Figure 4:**
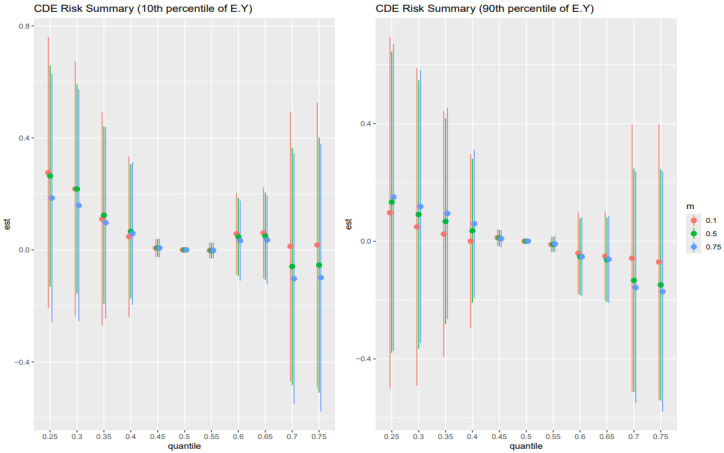
The controlled direct effect risk summary (at 10th and 90th percentiles) of liver disease outcome was examined at 0.25–0.75 quantiles of exposure.

Figure 4 shows the point estimates (dots) and 95% credible intervals (vertical bars) for the controlled direct effect (CDE) of exposure on the outcome, evaluated at the 10th percentile of E[Y] (younger adults, left panel) and the 90th percentile of E[Y] (older adults, right panel), with AL fixed at its 10th (red), 50th (green), and 75th (blue) percentiles across exposure quantiles.

In the older adult stratum (right panel), these patterns are most pronounced: CDEs start high and positive at low exposure (25th percentile), amplified by higher AL (blue > green > red), then decline steeply toward zero by mid-exposure, cross negative by the 75th percentile, and reverse ordering (low AL yields the least negative estimate). The younger adult stratum (left panel) shows the same overall shape but with smaller magnitudes and less stark separation by AL. Wide intervals at the extremes reflect substantial posterior uncertainty.

**Figure 5:**
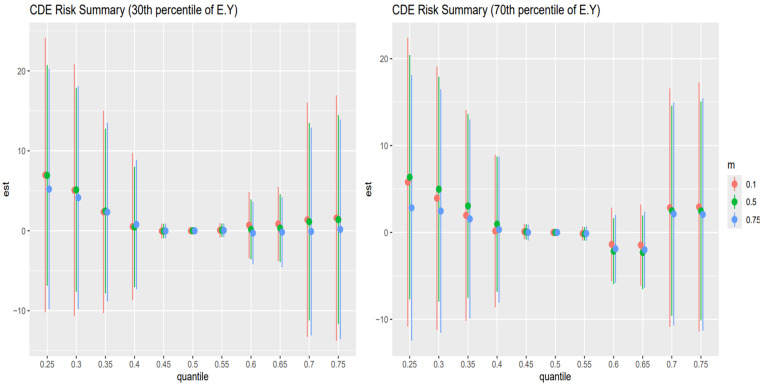
The controlled direct effect risk summary (at 30th and 70th percentiles) of liver disease outcome was examined at 0.25–0.75 quantiles of exposure.

Figure 5 shows the point estimates (dots) and 95% credible intervals (vertical bars) for the controlled direct effect (CDE) of exposure on the outcome, evaluated at the 30th percentile of E[Y] (younger adults, left panel) and the 70th percentile of E[Y] (older adults, right panel), with AL fixed at its 10th (red), 50th (green), and 75th (blue) percentiles across exposure quantiles.

In the older adult stratum (right panel), CDEs are largest and positive at low exposure quantiles (25th–35th), but here the mid-level AL (green) yields the highest effects, followed by low AL (red) and then high AL (blue). All three estimates decline sharply toward zero by the mid-range exposures (45th–55th percentiles) and cross negative by the 65th–75th percentiles, and the ordering reverses (high AL now most negative, low AL least negative). The younger adult stratum (left panel) mirrors this shape—positive CDEs at low exposures with green > red > blue, fading through zero to negative at the top quantiles—but with smaller magnitudes and less separation by AL. Wide intervals at the extremes reflect substantial posterior uncertainty.

### 3.6. Single-Predictor Risk Summary on Allostatic Load Across Exposure Quantiles: Metals and Lifestyle Habits

**Figure 6:**
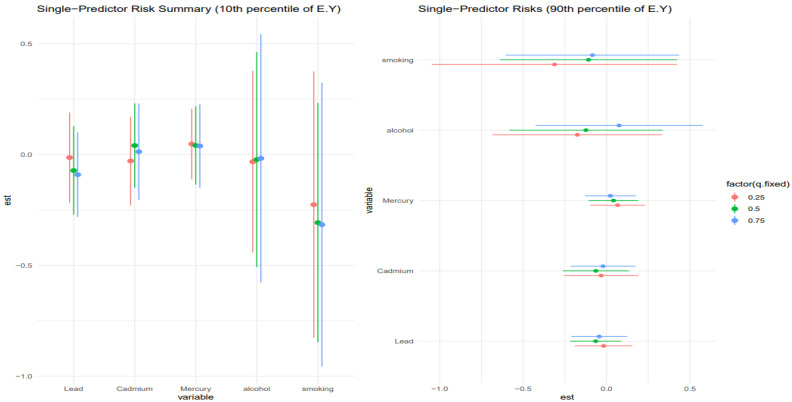
Single-exposure effect of the individual exposure on FLI; examining the change in response associated with a change in a single exposure from its 25th to 75th quantile while all other exposures are fixed at a specific quantile (25th, 50th, and 75th). Results shown for the 10th (**left**) and 90th (**right**) percentiles for age.

Figure 6 shows the single predictor effect and 95% credible interval, defined as the change in response associated with a change in a single exposure from the 0.25 to 0.75 quantile, with all other exposures fixed at a specific quantile, 0.25 (red), 0.50 (green), and 0.75 (blue). This is done for those at the 10th percentile of age and the 90th percentile for age. Results indicate that for the 10th percentile of age, the effects were largely zero, apart from smoking, which had a negative effect. The chart also gives insight into interactions, with smoking and lead showing signs of interaction. For the 90th percentile of age, the results were similar, with smoking and alcohol showing the most interaction.

**Figure 7:**
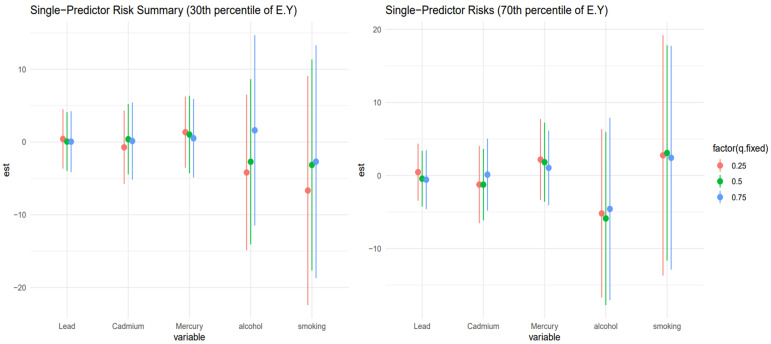
Single-exposure effect of the individual exposure on FLI; examining the change in response associated with a change in a single exposure from its 25th to 75th quantile while all other exposures are fixed at a specific quantile (25th, 50th, and 75th). Results shown for the 30th (**left**) and 70th (**right**) percentiles for age.

Figure 7 shows a similar analysis to Figure 6 but examines the 30th percentile and 70th percentile for age. Results for the 30th percentile of age were largely around zero, with alcohol and smoking showing the most interaction. For the 70th percentile of age, smoking had the largest effect, but the large credible intervals revealed the uncertainty in the results. For the 70th percentile, there was little single-variable interaction.

### 3.7. Mediation Effects

BKMR-CMA was also used to estimate the direct and indirect effects of heavy metals and behavioral habits on FLI with AL as a mediator. Different colors represent the percentiles; the red color represents the younger age group, while the blue color represents the older age group.

**Figure 8:**
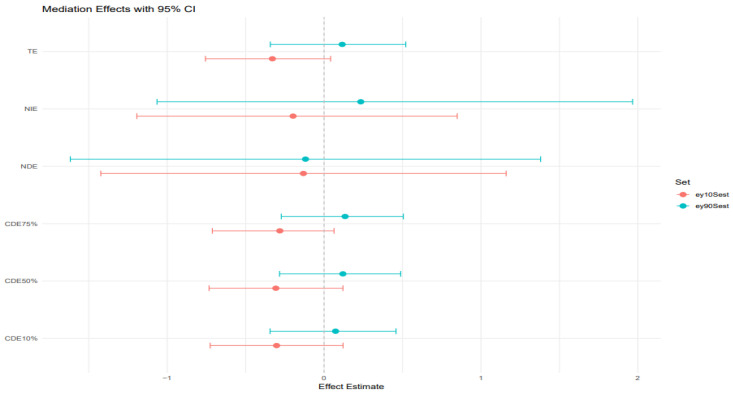
Mediation analysis assessing the combined effects of heavy metals and behavioral factors on FLI with AL as mediator at ages 10th and 90th percentiles.

Figure 8 plot: 10th and 90th percentile of age.

TE (Total effect): The total effect, which includes both the direct and indirect effects, is slightly positive and above zero for the 10th percentile age group but significantly negative for the 90th percentile age group. The credible interval is slightly wide for both age groups, indicating uncertainty in the impact of heavy metals and behavioral habits on FLI.

NIE (Natural indirect effect): This represents the portion of the exposure effect on FLI that is mediated through AL. The estimate is close to zero but still positive for the younger age group and negative for the older age group. They both have sizeable credible intervals crossing zero, indicating the results have some uncertainty.

NDE (Natural direct effect): The natural direct effect, independent of AL, is close to zero and negative for both age groups. They both have large credible intervals crossing zero as well. CDEs at different quantiles (10%, 50%, 75%): The controlled direct effects show slightly positive estimates for the younger individuals but negative for the older individuals. The credible intervals for both age groups are narrower but cross zero, indicating that while there may be a weak positive association with AL in the younger individuals, the results remain highly uncertain and should be interpreted cautiously.

**Figure 9:**
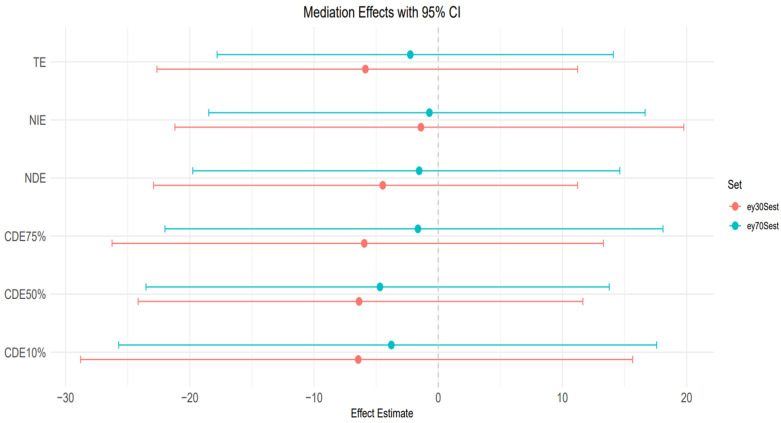
Mediation analysis assessing the combined effects of heavy metals and behavioral factors on FLI with AL as mediator at ages 30th and 70th percentiles.

Figure 9 plot: 30th and 70th percentile of age.

TE (Total effect): The total effects for both age groups (30th and 70th percentiles) taper slightly below zero and become negative. Also, the credible interval is large and crosses zero, suggesting substantial uncertainty.

NIE (natural indirect effect): NIE is also negative for both age groups, and the credible interval is significantly wide and indicating uncertainty.

NDE (Natural direct effect): For both age groups, the NDE is negative with a large credible interval crossing zero, indicating uncertainty in the estimates.

CDEs at different quantiles (10%, 50%, 75%): The controlled direct effect shows a slight downward trend and a negative trend across the AL quantiles for both age groups. All credible intervals are relatively wide and cross zero, which indicates the uncertainty in the results for both groups.

## 4. Discussion

This study examined how heavy metal exposures and behavioral risk factors interact to influence liver health, with AL, a measure of cumulative physiological stress, as a mediating role. By combining linear regression with BKMR-CMA, we investigated both direct and indirect pathways through which lead, cadmium, mercury, smoking, and alcohol might affect liver biomarkers and overall hepatic function. Our findings contribute to a more nuanced understanding of how chronic stress physiology intersects with environmental and behavioral exposures in causing liver disease risk.

Results from the linear regression analysis revealed that lead was the most consistent environmental predictor of liver injury, showing a statistically significant positive association with AST and a marginal association with ALT. This suggests that lead exposure may be linked to hepatocellular damage, a result that is biologically plausible given lead’s known role in promoting oxidative stress, disrupting mitochondrial function, and impairing hepatic detoxification systems [28,29]. These enzyme elevations, while modest in effect size, align with previous research linking lead exposure to subclinical liver injury and support the need for monitoring even low-level lead burdens in at-risk populations [30].

In contrast, cadmium and mercury did not show statistically significant associations with liver enzymes, though mercury exhibited a borderline association with FLI. This may suggest a more subtle or delayed role in metabolic dysfunction, consistent with findings from studies implicating mercury in lipid metabolism and insulin resistance, which is likely mediated through increased oxidative stress, inflammation, and possible mitochondrial dysfunction [31,32]. Moreover, our age-stratified descriptive data revealed notable trends; lead levels increased progressively with age, peaking in the 70–90 age group, reflecting its cumulative nature and long half-life. Mercury levels were most variable in the 60–70 age group, which could correspond with dietary habits such as seafood consumption [33]. Cadmium, on the other hand, exhibited greater variability in younger individuals likely due to differential smoking behavior and occupational exposures, highlighting lifestyle’s role in modulating environmental toxin burden.

Among behavioral exposures, smoking emerged as a prominent contributor to liver enzyme alterations. Smoking was positively associated with GGT and ALP enzymes linked to oxidative stress and biliary tract dysfunction [34]. These results reinforce the established view that smoking exacerbates hepatic oxidative damage and impairs bile processing. Interestingly, smoking was also inversely associated with total bilirubin, a potential indicator of impaired bilirubin metabolism or increased oxidative demand, since bilirubin also functions as an endogenous antioxidant [35,36]. The impact of alcohol consumption, while expected to be significant, was less consistent. Alcohol did not reach statistical significance for most liver enzymes but was negatively associated with FLI, which contradicts conventional assumptions. This surprising result may reflect confounding due to self-report bias, underreporting of alcohol intake among health-compromised individuals, or reverse causality in which individuals with known liver disease reduce alcohol use [37,38].

AL, simply known as the cumulative burden of chronic stress on physiological systems, was a key focus of this study [39]. AL was strongly associated with FLI (*p* < 0.001), indicating that chronic stress contributes significantly to hepatic fat accumulation and potentially to the pathogenesis of metabolic liver diseases such as MAFLD [40]. This association held even after adjusting for conventional risk factors such as BMI, income, and age, suggesting that stress physiology plays a role independent of metabolic syndrome components.

Furthermore, AL showed a near-significant association with ALP and total bilirubin, suggesting potential links to bile duct stress or hepatic excretory function. However, AL was not significantly associated with AST, ALT, or GGT in multivariable models. This pattern suggests that AL may be more relevant to metabolic dysfunction and long-term stress-related hepatic changes rather than acute liver injury or inflammation [40,41].

From our descriptive analysis, AL peaked in the 50–60 age group, consistent with prior research suggesting that middle age represents a period of heightened stress accumulation [42]. Moreover, gender and racial/ethnic differences in AL distribution were observed, with women and Mexican Americans exhibiting higher levels of AL, suggesting that socioeconomic and demographic disparities may amplify physiological stress burdens.

The estimated total effects of the combined exposure mixture, including heavy metals (lead, cadmium, mercury) and behavioral risk factors (alcohol consumption and smoking), on fatty liver index (FLI), as modeled using BKMR, demonstrate age-stratified heterogeneity that reflects both nonlinear dose–response dynamics and age-specific physiological vulnerability. These patterns, depicted in Figure 2 and Figure 3, suggest that the biological impact of co-exposure to toxicants and behaviors is not static across the life course, but rather shaped by interactions between age, exposure burden, and latent stress-mediated pathways [43].

Among the youngest individuals (10th percentile of age, Figure 2 left panel), total effect estimates showed a modest positive trend at lower exposure quantiles that dissipate around the median and become slightly negative at higher exposure levels. Although the credible intervals are wide, reflecting uncertainty due to smaller subgroup sizes or latent variation, this subtle inverted-U shape hints at an early vulnerability threshold where low-to-moderate levels of combined exposures may begin to perturb hepatic metabolic processes before adaptation or protective responses mitigate further damage at higher doses [44]. These findings may be explained by developmental differences in liver detoxification capacity and the immature regulation of stress-response systems, which could render younger individuals more susceptible to subclinical hepatic dysfunction from modest toxicant-behavior interactions [45]. However, the decline in effect at higher exposures could reflect the limited cumulative burden of stress and toxicants at this life stage or compensatory physiological mechanisms still intact in younger livers [46,47].

In contrast, older adults (90th percentile of age, Figure 2 right panel) exhibited a different profile: effect estimates began near zero at low quantiles and progressively declined as exposure increased. This trend suggests that among the elderly, higher cumulative exposure to metals and behavioral risks may suppress liver function or worsen hepatic outcomes, consistent with the concept of physiological weathering. As individuals age, the cumulative toll of oxidative stress, inflammatory dysregulation, and diminished liver regenerative capacity may reduce resilience to toxicants. The downward-sloping curve implies that even in the presence of low AL or stress levels, the accumulated biological burden of aging heightens the risk of liver dysfunction in response to environmental–behavioral exposures [48]. However, wide credible intervals again caution against overinterpretation, indicating the need for replication and stratified model optimization in larger datasets.

The intermediate age groups, 30th and 70th percentiles (Figure 3), present more complex, less monotonic dose–response curves, pointing to a transitional window of toxicological vulnerability. For those at the 30th percentile (Figure 3, left panel), effect estimates initially decline toward the null at mid-exposures but rise slightly again at higher quantiles, suggesting a mild rebound effect. This may reflect the early stages of exposure accumulation in mid-adulthood, where the effects of toxicant-behavior combinations are emerging but still modulated by robust hepatic function [49]. Alternatively, this pattern may result from heterogeneous exposure histories or varying rates of metabolic adaptation across individuals in this group.

The 70th percentile age group (Figure 3, right panel) reveals a more erratic, non-monotonic pattern: the effect fluctuates from slightly positive to negative and back to positive again at the highest quantiles. This volatility, combined with wide credible intervals, may be driven by competing forces at this stage of life, where the body is no longer fully resilient to toxic stress, yet not as fully deteriorated as in the oldest cohort. The inconsistency in direction may also be shaped by differential interaction effects between exposures, AL, and latent comorbidities that tend to accumulate in later life [50,51]. Importantly, the lack of a stable trend suggests that the mixture effect becomes less predictable in the aging process, emphasizing the importance of modeling both mediation and interaction effects in these populations.

Collectively, these results suggest that the total effect of metal and behavioral mixtures on liver disease is nonlinear, age-dependent, and marked by a high degree of posterior uncertainty. The general shape of the exposure–response curves supports the idea that hepatic vulnerability is not linearly tied to exposure burden; rather, it is mediated by life-course stress biology, resilience capacity, and potentially compensatory mechanisms that differ across age groups. From a mechanistic standpoint, these findings align with the emerging literature on the exposome and biological aging, which emphasizes cumulative, interacting effects of social, behavioral, and chemical stressors over time [52].

The analysis of controlled direct effects across age strata revealed complex, nonlinear associations between toxicant exposures and liver outcomes. In both the youngest (10th percentile) and oldest (90th percentile) age groups (Figure 4), CDEs exhibited an inverted U-shaped pattern, with positive effects at lower exposure levels that diminished and, in some cases, reversed direction at higher exposures. Importantly, in this analysis, AL was specified solely as a mediator. The CDEs therefore represent the estimated effect of exposures on liver outcomes when AL is fixed at a given level, rather than any modifying (interaction) influence of AL. These findings highlight the complexity of direct exposure–outcome relationships and emphasize the importance of distinguishing mediation from effect modification when interpreting BKMR-CMA results [53,54]. Among younger individuals, the same shape persisted but with attenuated magnitudes and minimal separation by AL, suggesting greater physiological resilience and stress-buffering capacity in early life.

This dynamic was further supported by findings from the 30th and 70th percentiles of age (Figure 5), which revealed similar nonlinear trajectories but introduced important nuances. In these groups, mid-level AL (50th percentile) was often associated with the largest CDEs at low exposure levels, while both low and high AL attenuated these effects, particularly in midlife. This non-monotonic pattern may indicate a threshold effect, where moderate stress enhances vulnerability by temporarily sensitizing stress-response pathways, whereas chronic high stress induces adaptive downregulation or biological blunting. Across all age strata, the convergence of CDE estimates around the mid-range of exposure and their eventual reversal at the upper quantiles, especially in older adults, suggests a ceiling effect in which the liver’s capacity to metabolize and respond to toxicants potentially becomes impaired, possibly through cumulative damage or disruption of compensatory mechanisms such as antioxidant defense or HPA axis regulation [55,56]. These findings align with theoretical models of AL and stress toxicology, in which physiological responses to environmental insults are shaped by both cumulative stress burden and life-course stage.

Taken together, these results provide compelling evidence that the toxicological impact of metal and behavioral exposures on liver health is not fixed but is strongly contingent upon both age and stress burden. The identification of consistent AL-based effect modification, particularly in older individuals, underscores the relevance of stress physiology as both a mediator and a modifier in environmental health pathways. These patterns have important implications for public health strategies aimed at reducing liver disease risk in vulnerable populations: interventions targeting either toxicant exposure or chronic stress may yield synergistic benefits, especially among older adults, where resilience is diminished and the compounding effects of exposures and stress are most evident.

Figure 6 and Figure 7 presented the single-exposure effect estimates and their corresponding 95% credible intervals across different age strata, helping to disentangle the role of individual exposures, specifically smoking, alcohol, and heavy metals (e.g., lead), in driving liver disease risk. These analyses are critical for understanding how the toxicological and behavioral burden interacts with biological aging and stress system dysregulation, especially in the context of chronic liver injury and dysfunction.

Figure 6 stratified results by the 10th and 90th percentiles of age, capturing the youngest and oldest participants in the sample. For the youngest age group (10th percentile), most single predictor effects hovered around zero, except for smoking, which demonstrated a consistent negative effect on liver outcomes. This finding may reflect early vulnerability to behavioral exposures, potentially due to still-developing metabolic systems and stress regulatory networks [57,58]. Moreover, observed indications of interaction between smoking and lead are noteworthy, although not statistically robust given overlapping credible intervals, as these trends suggest potential synergistic hepatotoxicity, possibly mediated via oxidative stress and inflammation pathways that become dysregulated even at younger ages under dual exposure conditions.

Among individuals at the 90th percentile of age, results were broadly similar in that smoking and alcohol again emerged as the dominant contributors, with other exposures (e.g., lead, cadmium, mercury) having more muted effects. However, in this older subgroup, the interactions appeared amplified, particularly for alcohol and smoking, which may reflect age-related declines in hepatic resilience and a heightened susceptibility to cumulative toxicant burden. This is consistent with known aging processes such as reduced hepatic detoxification capacity, increased baseline inflammation, and dysregulation of the hypothalamic–pituitary–adrenal axis, all of which could increase vulnerability to liver injury through stress-related biological pathways [46,59].

Figure 7 extends this analysis to participants at the 30th and 70th percentiles of age, representing mid-life and early older adulthood. Among those at the 30th percentile, estimated effects for most exposures remained close to zero, but smoking and alcohol again displayed notable signals, suggesting that behavioral exposures begin to exert measurable hepatic impacts earlier in life, even when toxic metal exposures are minimal. Importantly, interactions were more evident at this age level, which may indicate a transitional window where exposure synergies begin to materialize but biological compensation mechanisms are still somewhat intact.

In contrast, the 70th percentile group revealed smoking as the most influential single exposure, but credible intervals were wide, indicating substantial posterior uncertainty. This is a common challenge in age-stratified mixture modeling where sample sizes decline, and variance inflates at tails of the age distribution. Notably, there was little evidence of single-variable interactions in this group, which could reflect a flattening or saturation of the exposure–response function at older ages. That is, once hepatic stress systems are maximally taxed (or already impaired), additional interactions between exposures may yield diminishing marginal effects, a concept consistent with theories of physiological ceiling effects in aging populations [60,61].

### The Mediating Role of Allostatic Load

To assess whether AL mediates the effects of environmental metals and behaviors on liver outcomes, we employed BKMR-CMA, a robust method capable of handling complex interactions and nonlinearities. The mediation models did not yield statistically significant indirect effects via AL. Credible intervals for the natural indirect effects (NIEs) consistently included zero, indicating uncertainty in AL serving as an intermediary between exposures and liver biomarkers in this sample. However, this uncertainty does not preclude biological relevance, particularly given the small sample size (*n* = 308), potential measurement error in AL components, and the complexity of the hypothesized pathways.

The BKMR-CMA results suggest that AL may serve as a modest mediator in the relationship between combined exposures to heavy metals and behavioral factors and liver disease risk. Notably, the natural indirect effect appears more pronounced in the older age group (90th percentile of age), suggesting that AL plays a stronger mediating role in older individuals. This may reflect increased physiological vulnerability to stress-related dysregulation with aging [62,63]. The credible intervals are wide and overlap with zero, but the directional trends are consistent with existing literature (e.g., Juster et al. [64]), which shows that cumulative stress burden amplifies age-related susceptibility to metabolic and hepatic dysfunction. These findings highlight the potential importance of age as an effect modifier in the pathway linking environmental and behavioral exposures to liver disease, acting through stress-mediated physiological wear and tear [40].

Although our findings also strongly suggest AL functions as a potent independent risk factor. The linear regression analysis provided the clearest evidence for its independent role, revealing a highly significant and strong positive association between AL and FLI (β = 3.66, *p* < 0.001). This indicates that for every one-unit increase in the AL score, the FLI score increased by 3.66 points, highlighting chronic physiological stress as a major contributor to hepatic fat accumulation, irrespective of the other exposures.

The similarity of Controlled Direct Effects across AL percentiles for both the 10th and 90th percentiles of age (Figure 8) suggests that AL does not modify the direct effect of environmental and behavioral exposures on liver disease in the youngest and oldest segments of the population. Instead, AL appears to act more clearly as a mediator, transmitting part of the exposure’s effect on hepatic dysfunction via stress-related physiological wear.

However, the CDE patterns at the 30th and 70th percentiles of age (Figure 9) reveal greater instability and wider variability across AL levels, particularly in the individuals around the 70th percentile of age. In this group, the Controlled Direct Effects exhibit more noticeable deviation, implying potential effect modification by AL. This suggests that in midlife or early older adulthood, the influence of stress burden may interact with exposures to alter liver disease risk, pointing to a complex interplay between age, stress, and toxicant susceptibility. Nevertheless, the wide credible intervals that include zero suggest uncertainty of these findings. However, AL’s influence in regression analysis was less clear for traditional liver enzymes (ALT, AST, ALP, GGT), which displayed highly variable effects depending on exposure combination. These enzymes may reflect more acute hepatic responses to toxicants, whereas FLI is more indicative of longer-term metabolic changes, aligning better with AL’s chronic, systemic nature.

The differential patterns across liver biomarkers suggest that AL influences some hepatic outcomes more than others, potentially due to differences in temporal onset, biological pathway, or measurement sensitivity. This complexity underscores the importance of distinguishing between immediate hepatocellular injury and chronic liver metabolic burden when examining environmental and psychosocial risk factors.

Moreover, previous studies have noted that interventions aimed at reducing AL, such as mindfulness training, social support, and physical activity, can improve overall metabolic health but often fall short in reversing damage caused by persistent environmental toxicant exposure [65,66]. Our results echo this perspective: AL may exacerbate the harm caused by heavy metals and smoking, but it may not be sufficient on its own to fully mediate or counteract these exposures.

Taken together, our findings reveal that liver dysfunction is the product of multidimensional stressors, involving both exogenous exposures (pollutants, behaviors) and endogenous responses (physiological burden, metabolic dysregulation). The strongest and most consistent associations were observed between lead and liver enzymes, smoking and cholestatic indicators, and AL and metabolic liver outcomes. While AL did not emerge as a dominant mediator, its significant direct association with FLI and its interaction with age and behavioral stressors suggest that it should not be overlooked in liver disease etiology.

Study Limitations

This study is limited by its cross-sectional design, which restricts causal inference and prevents establishing temporal order between exposures, stress, and liver outcomes. While BKMR-CMA allowed us to explore complex exposure–mediator–outcome relationships, its use with cross-sectional data should be interpreted cautiously, as the assumptions required for causal mediation cannot be fully verified without longitudinal data. Additionally, the sample size might have limited the precision of some estimates, particularly in stratified or age-specific BKMR models, which may explain wider credible intervals observed in certain analyses. In addition, the use of self-reported behavioral data, such as alcohol intake and smoking may have introduced measurement error. Inaccurate recall or the tendency to provide socially acceptable responses by participants could result in misclassification of exposure status, thereby weakening the observed associations between behavioral factors and liver outcomes.

Future Directions

Future studies should consider using longitudinal data to better capture how chronic exposures and stress evolve over time and how they contribute to liver disease development. Following participants over several years would help clarify whether AL truly acts as a mediator or more as a modifier in the pathway between environmental exposures and liver outcomes.

It would also be beneficial to refine the measurement of AL. Including a broader range of biomarkers, such as cortisol or inflammatory markers, could give a more accurate picture of physiological stress. Additionally, using exposure measures that reflect cumulative or long-term body burden, such as bone lead or urinary cadmium, would better align with the chronic nature of liver disease. Further studies could also explore how AL interacts with genetic or epigenetic predispositions to liver disease, which may help uncover more specific patterns of vulnerability across different individual risk profiles. This could help identify subgroups of people who are not only exposed to environmental and behavioral risks but are also genetically more vulnerable to stress-related liver damage. Additionally, although NHANES collects physical activity data, it was not included in this analysis to maintain model parsimony; however, future studies should explore its potential modifying role, particularly among older adults.

Lastly, age stands out as an important factor in our study. Future research should take a life course approach to better understand when individuals are most vulnerable to combined exposures and stress, whether during childhood, midlife, or later in adulthood.

## 5. Conclusions

This study offers compelling evidence that liver health is shaped not only by individual toxicants or behaviors but by their combined burden and the biological stress responses they invoke. While lead exposure and smoking were independently associated with elevations in key liver enzymes, and AL was strongly linked to the FLI, the mediation analysis revealed that AL’s role as a mediator between exposures and liver damage was less mediating.

These findings underscore the importance of considering AL as both a biological reflection of cumulative life stress and a potential amplifier of environmental and behavioral factors. Although mediation was not statistically significant, the directional trends suggest that AL may still contribute meaningfully to liver disease pathogenesis, especially through chronic metabolic alterations rather than acute hepatocellular injury.

In summary, the study advances a systems-level perspective on liver health, emphasizing the need to assess multifactorial interactions across environmental, behavioral, and physiological domains. By recognizing the complex interplay of stress, toxins, and behaviors, this research paves the way for more holistic and integrated public health strategies to address liver disease and create interventions, especially in populations facing disproportionate exposure to both environmental toxins and chronic stress.

## Figures and Tables

**Table 1:** Lead by Age Decile.

Age Decile (Years)	Mean (µg/dL)	Std Dev	Min	Max	Count
10–20	0.97	0.33	0.68	1.34	5
20–30	1.01	1.04	0.15	7.36	47
30–40	0.94	0.61	0.29	3.91	62
40–50	2.08	5.62	0.25	42.48	55
50–60	1.75	1.02	0.48	6.29	68
60–70	2.26	1.99	0.64	12.71	54
70–80	3.05	2.11	0.89	8.26	13
80–90	2.71	0.63	2.04	3.42	4

**Table 2:** Cadmium by Age Decile.

Age Decile (Years)	Mean (µg/dL)	Std Dev	Min	Max	Count
10–20	1.27	0.44	0.57	1.7	5
20–30	1.13	0.80	0.07	3.92	47
30–40	1.18	0.92	0.15	4.6	62
40–50	1.17	0.80	0.1	3.86	55
50–60	1.31	0.98	0.27	5.13	68
60–70	1.35	0.98	0.35	4.55	54
70–80	1.33	0.77	0.36	3.25	13
80–90	1.06	0.11	0.93	1.19	4

Mercury was highly variable, particularly in middle-aged and older individuals. The 60–70 age group exhibited the highest mean (1.44 µg/L) and maximum value (20.53 µg/L). The results are presented in Table 3 below.

**Table 5:** Frequency distribution of categorical variables.

Variable/Category	AL Binary	Frequency	Percentage (%)
**Sex**			
Female	≤3	58	46.77
Female	>3	66	53.23
Male	≤3	103	55.98
Male	>3	81	44.02
** Race/Ethnicity **			
Mexican American	≤3	8	26.67
Mexican American	>3	22	73.33
Non-Hispanic Asian	≤3	8	44.44
Non-Hispanic Asian	>3	10	55.56
Non-Hispanic Black	≤3	67	65.69
Non-Hispanic Black	>3	35	34.31
Non-Hispanic White	≤3	59	50.86
Non-Hispanic White	>3	57	49.14
Other Hispanics	≤3	6	46.15
Other Hispanics	>3	71	53.85
Other Races	≤3	13	44.83
Other Races	>3	16	55.17

**Table 6:** Regression results for AST.

Variable	Coef.	Std. Err.	t	*p* > |t|	[0.025]	[0.975]	Std. Residuals
Constant	27.36	6.38	4.29	0.00	14.80	39.92	14.56
Lead	0.65	0.33	1.99	0.04	0.01	1.29	14.56
Cadmium	0.62	1.03	0.60	0.55	−1.41	2.65	14.56
Mercury	−0.59	0.52	−1.12	0.26	−1.61	0.44	14.56
Alcohol	−0.30	0.29	−1.02	0.31	−0.88	0.28	14.56
Smoking	−0.11	0.11	−0.97	0.33	−0.33	0.11	14.56
AL	−0.40	0.58	−0.69	0.49	−1.55	0.74	14.56
Age	0.03	0.06	0.57	0.57	−0.08	0.15	14.56
Gender	−1.74	1.86	−0.94	0.35	−5.40	1.91	14.56
Ethnicity	0.38	0.59	0.65	0.52	−0.78	1.54	14.56
Income	−0.06	0.05	−1.36	0.17	−0.15	0.03	14.56
Education	1.67	0.89	1.87	0.06	−0.09	3.42	14.56
BMI	−0.20	0.12	−1.66	0.09	−0.44	0.04	14.56

**Table 7:** Regression results for ALT.

Variable	Coef.	Std. Err.	t	*p* > |t|	[0.025]	[0.975]	Std. Residuals
Constant	24.89	6.85	3.64	0.00	11.43	38.37	15.62
Lead	0.66	0.35	1.88	0.06	−0.03	1.34	15.62
Cadmium	1.11	1.11	0.99	0.32	−1.08	3.29	15.62
Mercury	−0.03	0.56	−0.06	0.95	−1.13	1.07	15.62
Alcohol	−0.30	0.31	−0.95	0.34	−0.92	0.32	15.62
Smoking	−0.18	0.12	−1.53	0.13	−0.42	0.05	15.62
AL	0.34	0.62	0.54	0.59	−0.89	1.56	15.62
Age	−0.06	0.06	−0.95	0.34	−0.19	0.07	15.62
Gender	−9.21	1.99	−4.63	0.00	−13.13	−5.29	15.62
Ethnicity	0.28	0.63	0.45	0.66	−0.96	1.52	15.62
Income	−0.08	0.05	−1.69	0.09	−0.18	0.01	15.62
Education	1.46	0.96	1.53	0.13	−0.42	3.34	15.62
BMI	0.34	0.13	2.58	0.01	0.08	0.59	15.62

Note: Gender was coded as 1 = male and 2 = female; thus, the negative coefficient for gender indicates lower ALT levels among females relative to males.

**Table 8:** Regression results for ALP.

Variable	Coef.	Std. Err.	t	*p* > |t|	[0.025]	[0.975]	Std. Residuals
Constant	52.95	11.62	4.56	0.00	30.09	75.81	26.50
Lead	0.44	0.59	0.74	0.46	−0.73	1.61	26.50
Cadmium	−2.35	1.88	−1.25	0.21	−6.05	1.36	26.50
Mercury	0.04	0.95	0.04	0.96	−1.83	1.91	26.50
alcohol	0.98	0.53	1.84	0.07	−0.07	2.03	26.50
smoking	0.78	0.20	3.81	0.00	0.38	1.18	26.50
AL	1.97	1.06	1.86	0.06	−0.11	4.05	26.50
Age	0.32	0.11	2.94	0.00	0.11	0.53	26.50
Gender	−1.42	3.38	−0.42	0.68	−8.07	5.23	26.50
Ethnicity	−0.83	1.07	−0.78	0.44	−2.93	1.28	26.50
Income	−0.00	0.08	−0.05	0.96	−0.17	0.16	26.50
Education	1.61	1.62	0.99	0.32	−1.58	4.80	26.50
BMI	−0.25	0.22	−1.11	0.27	−0.68	0.19	26.50

**Table 9:** Regression results for GGT.

Variable	Coef.	Std. Err.	t	*p* > |t|	[0.025]	[0.975]	Std. Residuals
Constant	4.81	19.98	0.24	0.81	−34.51	44.13	45.59
Lead	1.99	1.02	1.96	0.05	−0.01	4.00	45.59
Cadmium	−0.98	3.24	−0.30	0.76	−7.35	5.38	45.59
Mercury	1.94	1.63	1.19	0.24	−1.27	5.16	45.59
alcohol	−1.06	0.92	−1.15	0.25	−2.86	0.75	45.59
smoking	0.79	0.35	2.24	0.03	0.09	1.48	45.59
AL	0.92	1.82	0.51	0.61	−2.66	4.50	45.59
Age	0.36	0.19	1.95	0.05	−0.00	0.73	45.59
Gender	−10.99	5.81	−1.89	0.06	−22.43	0.46	45.59
Ethnicity	2.20	1.84	1.20	0.23	−1.42	5.83	45.59
Income	0.07	0.14	0.51	0.61	−0.21	0.35	45.59
Education	4.09	2.79	1.46	0.14	−1.41	9.58	45.59
BMI	0.13	0.38	0.33	0.74	−0.62	0.88	45.59

**Table 10:** Regression results for Total Bilirubin.

Variable	Coef.	Std. Err.	t	*p*>|t|	[0.025]	[0.975]	Std. Residuals
Constant	0.69	0.10	7.03	0.00	0.49	0.881515	0.22
Lead	0.01	0.01	1.32	0.19	−0.00	0.016442	0.22
Cadmium	−0.01	0.02	−0.51	0.61	−0.04	0.023182	0.22
Mercury	0.00	0.01	0.26	0.80	−0.01	0.017828	0.22
alcohol	−0.01	0.00	−1.45	0.15	−0.02	0.002344	0.22
smoking	−0.00	0.00	−2.59	0.01	−0.01	−0.001076	0.22
AL	−0.01	0.01	−1.92	0.06	−0.03	0.000385	0.22
Age	0.00	0.00	0.72	0.47	−0.00	0.002461	0.22
Gender	−0.08	0.03	−2.77	0.01	−0.13	−0.022713	0.22
Ethnicity	0.00	0.01	0.39	0.69	−0.01	0.021302	0.22
Income	−0.00	0.00	−0.92	0.34	−0.00	0.000727	0.22
Education	−0.00	0.01	−0.01	0.99	−0.03	0.026858	0.22
BMI	−0.00	0.00	−0.31	0.76	−0.00	0.003106	0.22

**Table 11:** Regression results for FLI.

Variable	Coef.	Std. Err.	t	*p*>|t|	[0.025]	[0.975]	Std. Residuals
Constant	−55.99	6.99	−8.01	0.00	−69.76	−42.23	15.96
Lead	0.11	0.36	0.32	0.75	−0.59	0.82	15.96
Cadmium	−0.76	1.13	−0.67	0.51	−2.98	1.47	15.96
Mercury	0.99	0.57	1.74	0.08	−0.13	2.12	15.96
alcohol	−1.16	0.32	−3.62	0.00	−1.80	−0.53	15.96
smoking	0.17	0.12	1.34	0.18	−0.08	0.41	15.96
AL	3.66	0.64	5.75	0.00	2.41	4.91	15.96
Age	0.29	0.07	4.43	0.00	0.16	0.42	15.96
Gender	−9.08	2.03	−4.46	0.00	−13.09	−5.08	15.96
Ethnicity	−0.36	0.64	−0.56	0.57	−1.63	0.90	15.96
Income	−0.09	0.05	−1.79	0.07	−0.19	0.01	15.96
Education	1.43	0.98	1.47	0.14	−0.49	3.36	15.96
BMI	3.47	0.13	25.99	0.00	3.21	3.73	15.96

## Data Availability

The NHANES dataset is publicly available online, accessible at https://wwwn.cdc.gov/nchs/nhanes/continuousnhanes/overview.aspx?BeginYear=2017 (accessed on 12 September 2025).
